# Preliminary Effectiveness of a Smartphone App to Reduce Depressive Symptoms in the Workplace: Feasibility and Acceptability Study

**DOI:** 10.2196/11661

**Published:** 2018-12-04

**Authors:** Mark Deady, David Johnston, David Milne, Nick Glozier, Dorian Peters, Rafael Calvo, Samuel Harvey

**Affiliations:** 1 Black Dog Institute Faculty of Medicine University of New South Wales Sydney Australia; 2 Medical Research Council Cognition and Brain Sciences Unit University of Cambridge Cambridge United Kingdom; 3 School of Systems, Management and Leadership Faculty of Engineering and IT University of Technology Sydney Australia; 4 Brain and Mind Centre, Central Clinical School Faculty of Medicine and Health University of Sydney Sydney Australia; 5 School of Electrical and Information Engineering University of Sydney Sydney Australia

**Keywords:** depression, workplace, mHealth, smartphone, eHealth, pilot

## Abstract

**Background:**

The workplace represents a unique setting for mental health interventions. Due to range of job-related factors, employees in male-dominated industries are at an elevated risk. However, these at-risk groups are often overlooked. *HeadGear* is a smartphone app–based intervention designed to reduce depressive symptoms and increase well-being in these populations.

**Objective:**

This paper presents the development and pilot testing of the app’s usability, acceptability, feasibility, and preliminary effectiveness.

**Methods:**

The development process took place from January 2016 to August 2017. Participants for prototype testing (n=21; stage 1) were recruited from industry partner organizations to assess acceptability and utility. A 5-week effectiveness and feasibility pilot study (n=84; stage 2) was then undertaken, utilizing social media recruitment. Demographic data, acceptability and utility questionnaires, depression (Patient Health Questionnaire-9), and other mental health measures were collected.

**Results:**

The majority of respondents felt *HeadGear* was easy to use (92%), easily understood (92%), were satisfied with the app (67%), and would recommend it to a friend (75%; stage 1). Stage 2 found that compared with baseline, depression and anxiety symptoms were significantly lower at follow-up (*t*_30_=2.53; *P*=.02 and *t*_30_=2.18; *P*=.04, respectively), days of sick leave in past month (*t*_28_=2.38; *P*=.02), and higher self-reported job performance (*t*_28_=−2.09; *P*=.046; stage 2). Over 90% of respondents claimed it helped improve their mental fitness, and user feedback was again positive. Attrition was high across the stages.

**Conclusions:**

Overall, *HeadGear* was well received, and preliminary findings indicate it may provide an innovative new platform for improving mental health outcomes. Unfortunately, attrition was a significant issue, and findings should be interpreted with caution. The next stage of evaluation will be a randomized controlled trial. If found to be efficacious, the app has the potential to reduce disease burden and improve health in this at-risk group.

## Introduction

Mental health conditions, and depression specifically, are leading causes of long-term disability globally [1,2]. Such disorders curtail and prohibit an individual’s participation in basic activities of life including work [3]. The workplace has a complex relationship with mental well-being, as it is associated with both positive (eg, life satisfaction, personal autonomy, and confidence) [4,5] and negative mental health outcomes (eg, strain, stress, injury, and illness) [6]. Harvey et al’s [7] model of psychosocial workplace risk factors highlights the complex relationship between work and the development of mental health problems as well as the potential for administering psychological interventions in the workplace. With recent Australian data indicating disability support payments for psychiatric conditions are on the rise with these conditions now being the leading cause of sickness absence [8], the development of effective interventions is a pertinent concern.

Due to the predominate role work has in individuals’ lives, the workplace is increasingly being recognized as presenting a unique opportunity for both prevention and treatment of mental ill-health [9]. Although work strain is present across all industries, certain job-related factors make the issue more pertinent in some. Employees in male-dominated industries (MDIs; ie, those in which ≥70% workers are male, eg, agriculture, construction, mining, manufacturing, transport, and utilities [10]) have been found to be at heightened risk of mental health conditions [11,12]. This is likely due to a combination of job-related factors (eg, seasonal employment fluctuations leading to job insecurity, remote or isolated locations and family separation, and highly competitive, high-pressure work environments) [12] and the sociodemographic features of the employees themselves (eg, alcohol and substance abuse, low mental health literacy, and low rates of help seeking [13,14]). Related to—and compounding—both these areas is a traditional male attitude and workplace culture that has historically valued concepts of *toughness*, stoicism, and self-reliance [15,16]. Despite this need, little work has been specifically aimed toward these at-risk employees, with conventional prevention programs being poorly utilized by—or tailored to—these groups [17].

Electronic health (eHealth) and specifically mobile health (mHealth; health care practices supported by internet or mobile phone technologies) provide an opportunity to overcome some of the barriers present in traditional approaches to prevention and treatment [18]. Recent evidence suggests such interventions have utility in improving mental health outcomes in general [19-21], whereas workplace reviews have found eHealth interventions are effective at improving workers’ psychological well-being, increase work effectiveness [22], and mental health and stress symptoms [23]. Although the dominant therapeutic approach in this area is cognitive behavioral therapy, there is increasing evidence that mindfulness and other approaches may hold distinct utility in this space [22-24]. Furthermore, the high rates of smartphone ownership increase the viability of mobile mental health care interventions [25]. However, this area is still in its infancy and little is known about the feasibility of such approaches in MDIs specifically.

Considering these findings and gaps in the knowledge base, we sought to develop a smartphone-based workplace intervention to reduce depressive symptoms and promote well-being, with a specific focus on MDIs. This paper presents a methodological framework, based on that of the Medical Research Council (MRC) [26]; elucidates the development and initial testing of the app; and details the 2-staged testing approach to finalizing the development of the program.

The aim of this study is to evaluate the usability, acceptability, feasibility, and preliminary efficacy of a newly developed app (*HeadGear*) designed to reduce depressive symptoms in an MDI working population.

## Methods

### Study Design

The model used to develop the app involved a process of research and analysis, development, implementation, and evaluation. In developing new technologies, it was important that the framework was systematic (clear steps following a logical order), systemic (all processes critical for success are incorporated), reliable (steps are clearly described so that they can be replicated by other designers in other projects), iterative (the cycle of analysis design development testing revision can be repeated a number of times), and empirical (data gathering is built into the process and decisions are made on the basis of data) [27]. The development process utilized a 3-step approach based on the intervention mapping protocol [28]. Similarly, processes have been used successfully for mHealth app-based interventions [29]. The predominate emphasis of this paper is that of the third step, as other steps have been reported elsewhere [30,31].

Employees in MDIs were specifically targeted. The process took place from January 2016 to April 2017. An interdisciplinary team of computer engineers, psychiatrists, psychologists, and design experts (user experience and graphic designers) collaborated in the design and development of the app.

### Step 1: Defining the Problem

Although the effectiveness of eHealth and mHealth technologies for treating moderate levels of mental ill-health in general and clinical populations has been established, less is known about workplace eHealth interventions and eHealth prevention. The problem led the team to conduct a series of systematic reviews and meta-analyses to determine the effectiveness of workplace interventions for common mental disorders (CMD) [32], workplace depression prevention [33], the use of eHealth for prevention of CMD in general populations [34], and the use of eHealth tools for CMD in the workplace [23].

A recent meta-analysis of work-based depression prevention programs found such programs to be encouraging, with a number of different types of work-based interventions, particularly those based on cognitive behavioral models, demonstrating an ability to reduce depressive symptoms on unselected working populations [9].

### Step 2: Participatory Engagement

To develop a relevant and engaging program, it is important to involve end users in participatory design and user experience research [35]. This stage comprised several components, including 6 focus groups (N=60) with industry partners and an in-depth survey of 1 specific industry partner (N=105). The findings of these are reported separately [30,31], and following feedback, we aimed to design and prototype an app that is engaging for men in the target workplaces.

### Step 3: Design and Pilot Testing

Building on the outcomes of the initial 2 steps, the app content and design were finalized. The pilot testing of the app involved a 2-stage approach to test both initial utility and acceptability (using an alpha version [acceptance testing] of the app) and feasibility (engagement and perceived usefulness to users) and preliminary efficacy (using a beta version [operational testing] of the app). There were several reasons for this approach. Primarily, the costs involved in the creation of such technology are considerable. During the participatory engagement step, no prototype was used to generate unbiased input. However, it was necessary to be able to make modifications based on this testing and the usability of the app. Subsequently, modifications were made to the app between the 2 (alpha or beta) stages of the design and pilot testing step to refine usability elements and to test preliminary efficacy.

### The App

**Figure 1:**
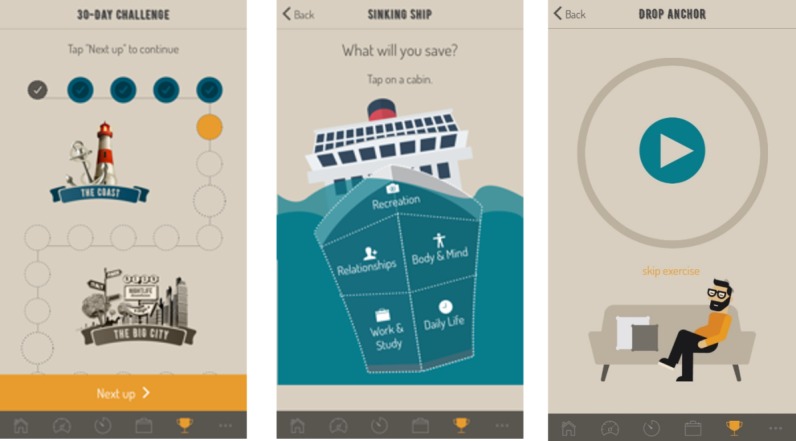
Intervention component of the *HeadGear* app: the 30-day challenge (left), a behavioral activation day (middle), and a mindfulness day (right).

*HeadGear* is a smartphone app–based intervention centered on behavioral activation and mindfulness therapy. The main therapeutic component of the *HeadGear* app takes the form of a 30-day challenge in which users’ complete 1 *challenge* daily (approximately 5-10 min; Figure 1). These *challenges* include psychoeducational videos on coping skills or resiliency, mindfulness, and behavioral activation; mindfulness exercises; value-driven activity planning, goal-setting, and review; and coping skill development (problem solving, sleep, grounding, alcohol use, assertiveness, and training in adaptive forms of coping). The inclusion of these specific components was driven by the findings of stages 1 and 2 (specifically, [9,23,30,31,34]).

**Figure 2:**
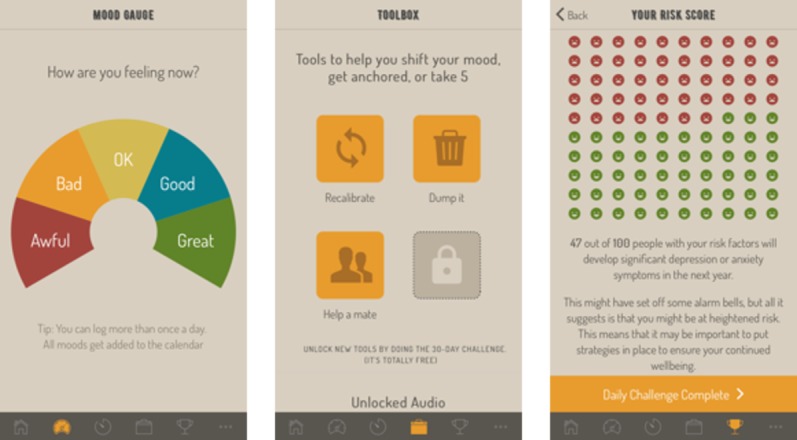
Additional features of the *HeadGear* app: mood widget (left), toolbox (middle), risk feedback (right).

The first daily challenge involves the completion of a risk calculator, which assesses and provides participants with personalized feedback regarding their risk for future mental health issues. The risk calculator consists of 20 items developed from the Household, Income and Labour Dynamics in Australia Survey (HILDA) and has been validated in the Australian adult population [36]. The risk factor items are based on participant self-report. The HILDA risk items include age, gender, Aboriginal and Torres Strait Islander status, active career status, freedom to decide work, satisfaction with hours worked, satisfaction with employment opportunities, physical activity, alcohol use, episodes of distress in the previous 2 years, satisfaction with health, satisfaction with the neighborhood, satisfaction with partner, satisfaction with the way tasks are divided with partner, having someone to confide in, the feeling of being pushed around, and English as a second language. The HILDA questions and response items were replicated from the original items included in the HILDA survey, apart from age, which is measured here as a continuous measure. Users received personalized risk feedback immediately after completing the risk calculator. The personalized risk feedback involves an interactive icon array, which displays the calculated numerical risk estimate of developing anxiety and depression within the next year, along with a text description (Figure 2). Although much of the app is not specific to a workplace (or even MDI) setting and is likely to have utility to a general population, it was within these populations that development occurred. The outcomes of early development work [30,31] led to the inclusion of certain elements, determined to be the most relevant among these groups. Importantly, the risk algorithm was built from a working population sample and was fundamental to its working population delivery.

Other components of the app include a mood monitoring widget, a toolbox of skills (which is built from the challenges as they are completed), and support service helplines. Users had access to the app indefinitely. The app monitors use time and frequency and mission completion rates.

### Stage 1: Alpha Testing—Utility and Acceptability

#### Participants

Participants (N=21) were recruited via email circulation and snowball recruitment from 3 industry partner organizations (agriculture, freight or postage, and mining). Study eligibility included Australian residency, aged between 18 and 65 years, valid email, ownership of an Apple- or Android-operating smartphone, ability to comfortably read English, and current employment. Consent was obtained electronically from all participants, and any identifiable data were encrypted to ensure confidentiality. The study acted in accordance with the Helsinki Declaration.

#### Procedure

Interested individuals were directed to the program’s website to undergo screening and provide informed consent via the Web-based participant information statement. Participants completed a baseline questionnaire and were then invited to download the app. As an alpha version, iPhone users were required to download the app via a third-party app *Testflight*. Participants were encouraged to use the app for 30 days as often as they wanted. At the end of this period, users completed a follow-up questionnaire within the app (with 2 reminder emails sent to noncompleters). Daily engagement in the intervention was not incentivized, but successful completion of the posttrial questionnaire placed participants in the draw for an Aus $300 gift voucher. The study was approved by the University of New South Wales (UNSW) Human Research Ethics Committee (HC No: 16646).

#### Measures

Participants completed self-administered questionnaires within the app. Demographic information provided included age, sex, education, occupation, role, location, and industry group. The follow-up survey comprised the same measures as in the initial battery with the addition of a 26-item acceptability and usability questionnaire (comprising adapted items from the System Usability Scale [37]; Post Study System Usability Questionnaire [38]; Technology Assessment Model Measurement Scales [39]; and Usefulness, Satisfaction, and Ease questionnaire [40]), and this blended tool has been used successfully in previous research [41]. Participants were asked to rate their agreement with a series of statements about the intervention. Usage data were automatically collected by the app including time spent in app, number of logins, and specific responses to exercises.

### Stage 2: Beta Testing—Feasibility and Preliminary Efficacy

#### Participants

Participants (N=84) were recruited via Facebook advertisements. All the advertisements were targeted (using Facebook’s advertising platform) to males aged between 18 and 65 years, located within Australia, and employed in an MDI. Facebook allowed targeting of the following industries: agriculture, engineering, transport, forestry, mining, plumbing, and construction. Inclusion eligibility criteria were the same as those in stage 1.

#### Procedure

The advertising campaigns all ran simultaneously between July and August 2017. Advertisements were restricted to be shown only on mobile devices. Clicking anywhere on the Facebook advertisement directed interested individuals to the study website where they completed consent electronically. Confidentiality was assured via data encryption. After giving consent, individuals were asked to provide a mobile phone number. This number was verified by sending it a short message service (SMS) text message containing a random 4-digit code, which the individual was required to enter on the study website to continue. After a successful verification, the individual was sent (via SMS text message) a link that allowed them to download the *HeadGear* app via the *Google Play* or *iOS* app store, depending on their device. Participants then proceeded to an in-app questionnaire that collected demographic information and contained a number of study-specific measures (see below). At 5 weeks post baseline, participants were sent a text message (with 2 reminder texts sent to noncompleters), which directed them to the study data-collection site, and responded to a similar questionnaire (with the removal of demographic items and inclusion of some program feedback questions). Postintervention assessment occurred at 5 weeks post baseline to allow users 1 extra week to complete the 30-day program.

Daily engagement in the intervention was not incentivized, but successful completion of the posttrial questionnaire placed participants in the draw for a Aus $200 gift voucher. The study was approved by the UNSW Human Research Ethics Committee (HC17021).

#### Measures

The Patient Health Questionnaire-9 (PHQ-9) was used to measure depression symptoms [42]. The PHQ-9 is a reliable and valid 9-item measure of depression severity over the past 2 weeks [42,43]. Each of the 9 items of the PHQ-9 is scored as 0 (not at all), 1 (several days), 2 (more than half the days), or 3 (nearly every day). As a screening tool, summing the 9 item leads to a maximum score of 27 indicating all symptoms occurring nearly daily. The criterion and construct validity of the PHQ-9 have previously been demonstrated, with 73% sensitivity and 98% specificity in detecting major depression compared with clinician-based assessment [42,44], and regardless of diagnostic status, it typically represents clinically significant depression [42]. The measure has demonstrated excellent internal consistency (Cronbach alpha >.85 in multiple samples) and test-retest reliability of .84 [43].

Anxiety was measured using the 2-item Generalized Anxiety Disorder (GAD-2) scale [45]. The GAD-2 consists of the 2 core criteria for generalized anxiety disorder, which have also been shown to be effective screening items for panic, social anxiety, and posttraumatic stress disorders [45]. Equivalent to the parent scales, the PHQ-2 begins with the following stem question: “Over the last 2 weeks, how often have you been bothered by the following problems?” Response options are “not at all,” “several days,” “more than half the days,” and “nearly every day,” scored as 0, 1, 2, and 3, respectively (total ranging from 0 to 6). Scale scores of 3 or above are suggested as cut-off points between the normal range and probable cases of anxiety [45].

Resilience was measured by the Connor Davidson Resilience Scale (CD-RISC), a 10-item self-report scale demonstrated to be psychometrically sound with high internal consistency (Cronbach alpha=.89), construct validity, and test-retest reliability in the general population and in clinical settings [46]. Total scores range from 0 to 40 with higher scores corresponding to greater resilience. Validity is highly relative to other measures and reflects differentiation in resilience among diverse populations, showing that higher levels of resilience are consistent with lower levels of perceived stress vulnerability [46]. The CD-RISC has been shown to differentiate between individuals who function well after adversity from those who do not and measures the core features of resilience and the ability to tolerate experiences [47]. It is believed that increased resilience may reduce rates of mental ill-health [48].

Well-being was assessed using the 5-item World Health Organization Well-Being Index (WHO-5) [49,50]. Raw scores range from 0 to 25, where 0 indicates the worst possible quality of life and a score of 25 represents the best possible quality of life. A score less than or equal to 13 or an answer of 0 or 1 on any of the 5 items shows poor well-being. WHO-5 is a psychometrically sound measure of well-being with high internal consistency (Cronbach alpha=.84) and convergent associations with other measures of well-being [51].

Work performance was assessed using a modified version of the World Health Organization Health and Work Performance Questionnaire (WHO-HPQ) [52]. The WHO-HPQ is a self-report instrument designed to estimate the workplace costs of health problems in terms of self-reported sickness absence and reduced job performance (presenteeism). The absolute presenteeism score derived from this tool ranges from 0 (total lack of performance during time on the job) to 100 (no lack of performance during time on the job) with higher scores indicating less presenteeism. Absolute presenteeism was calculated, given it has been associated with better construct validity than the relative measure [53].

The WHO-HPQ was modified to simplify the absenteeism measure. Short-term absenteeism was assessed by asking “how many days/shifts have you missed over the past 4 weeks (28 days) due to sickness absence.” If greater than 0, respondents were then asked, “how many of these sick days were due to mental health or emotional problems.” For long-term absenteeism it was asked, “over the last 6 months have you had a continuous 1-week period of sickness absence.” Following this question respondents were asked, “if yes, was this due to mental health or emotional problems?”

### Statistical Analysis

#### Sample Size

For stage 1, 25 individuals were sought to review the program. This number is not based on traditional power analysis calculations as our descriptive design precludes the ability to carry out power analyses. For this reason, we have drawn on previous studies in the field to guide in sample size determination.

Pilot studies (stage 2) tend to be underpowered to determine *proof-of-concept*. Additionally, the large sample size required for universal prevention work contribute to a lack of power in such pilot trials [54]. Despite this, for stage 2, using a 2-tailed test, with alpha set at *P*=.05 and power level of .80 (to detect a medium effect), a total of 40 participants was required. Due to expected high rates of dropout due to the unguided eHealth, general population, and nature of the study, an attrition rate of 50% was selected and a sample size of 80 was set.

#### Analysis Plan

All data were analyzed using IBM SPSS version 23.0 [55]. Stage 1 presents only descriptive statistics. In stage 2, descriptive statistics derived from participants’ smartphone use data were used to characterize engagement and acceptability in the pilot study. Paired sample *t* tests were used to test for differences between pre- and posttrial clinical outcomes (eg, PHQ-9). Symptom change scores were computed, and linear regression was performed to test for the effect of time spent using the app, level of baseline risk, or the industry of employment on symptom change. Standardized effect sizes (Cohen *d* [56]) were calculated for outcomes of interest following the methods reported in the study by Lipsey and Wilson [57].

## Results

### Stage 1: Alpha Testing

In total, 21 participants downloaded the app, 12 of whom responded to the follow-up survey. However, 6 participants consented but did not download the app and were subsequently removed from the study. The average age of the participants was 37.86 years (SD=10.98); approximately half of the participants were female (n=12). The majority of the sample worked in freight and postage (n=11), followed by mining (n=6) and agriculture (n=2); however, 3 participants declined to provide their industry. Approximately half of the participants were working in a manager role (n=9) and the majority were based in an urban center (n=15).

#### Utility

Participants on average completed 5.71 challenge days (SD=9.02) and logged an average of 3.33 (SD=5.48) moods. Participants were asked to rate their agreement with a series of statements about the app’s utility (see Table 1). Over 90% of participants reported that they believed most people would learn to use the app quickly and were satisfied with how easy the app was to use. Over 80% were comfortable using the app. The majority of negative feedback received came from 1 participant who only used the app to log 1 mood.

#### Acceptability

Table 2 shows respondents’ rating of the app’s acceptability. Over 90% of participants reported that they believed the information was easily understood and over 80% felt confident using the app. No respondent felt they needed to learn a lot of things before using the app. Over two-thirds of respondents were satisfied with the app, whereas 75% claimed it was fun to use, interactive, and that they would recommend it to a friend. Again, only 1 user reported substantial negative responses. There was a degree of concern about the utility of the app with only 40 to 50% of respondents claiming they would use it, or use it often, and 42% claiming the app worked the way they wanted it to. However, few actively disagreed with these statements.

**Table 1:** App utility questionnaire.

Statement	Disagree, n (%)	Neutral, n (%)	Agree, n (%)
I think that I would need the support of a technical person to be able to use the app	10 (83)	0 (0)	2 (17)
I found that the different parts of the app work well together	1 (8)	3 (25)	8 (67)
I thought there was too much inconsistency in the app	7 (58)	4 (33)	1 (8)
I would imagine that most people would learn to use the app very quickly	1 (8)	0 (0)	11 (2)
I found the app very awkward to use	10 (83)	0 (0)	2 (17)
Overall, I am satisfied with how easy it is to use the app	1 (8)	0 (0)	11 (92)
I was able to complete the “modules” quickly in the app	2 (17)	2 (17)	8 (67)
I felt comfortable using the app	1 (8)	1 (8)	10 (83)
Whenever I made a mistake using the app, I could recover easily and quickly	3 (25)	0 (0)	9 (75)
How things appeared on the screen was clear	1 (8)	2 (17)	9 (75)

**Table 2:** App acceptability questionnaire.

Statement	Disagree, n (%)	Neutral, n (%)	Agree, n (%)
I think that I would like to use the app often	1 (8)	6 (50)	5 (42)
I found the app to be very complicated	8 (67)	3 (25)	1 (8)
I felt very confident using the app	1 (8)	1 (8)	10 (83)
I needed to learn a lot of things before I could get going with the app	11 (92)	1 (8)	0 (0)
The information provided for the app was easy to understand	1 (8)	0 (0)	11 (92)
If I have access to the app, I will use it	1 (8)	5 (42)	6 (50)
I am satisfied with the app	1 (8)	3 (25)	8 (67)
I would recommend the app to a friend	1 (8)	2 (17)	9 (75)
The app is fun to use	1 (8)	2 (17)	9 (75)
The app helped me manage my symptoms	2 (17)	3 (25)	7 (58)
The app was interactive enough	1 (8)	2 (17)	9 (75)

**Table 3:** Demographics and app usage (N=84).

Characteristics		Statistics
Age in years, mean (SD)		38.62 (9.23)
Male, n (%)		84 (100)
Prior episode of mental ill-health, n (%)		48 (79)
Total active time in minutes, n (%)		58.24 (63)
Challenges completed, n (%)		9.11 (10)
Days used, n (%)		15.03 (16)
**Industry, n (%)**		
	Male-dominated industry^a^	37 (45)
	Nonmale dominated industry	45 (54)
	Industry not provided	2 (2)
**Role, n (%)**		
	General employee	55 (67)
	Manager	19 (23)
	Senior manager	8 (10)
	Role not provided	2 (2)
**Risk category [36], n (%)**		
	Low (≤4.5%; up to 25th percentile)	10 (12)
	Average (4.6%-22%; 25th to 90th percentile)	21 (251)
	High (≥23%; above 90th percentile)	30 (35)

^a^Agriculture or forestry or fishing, manufacturing, wholesale trade, mining, construction, other manual trade, transport or postal or warehousing, and first responder or defense or security.

#### Changes

A number of functionality and user interface and experience issues were resolved between stage 1 and 2. Additionally, changes were made to the app based on individual feedback. This included improved risk feedback (to better explain the feedback and direct users to elements in the challenge or external help), reminder functionality, a new booster session video added, goal-setting changes (to link values to both small and larger goals), and improvements to the skill toolbox (allowing for better integration with the challenge).

### Stage 2: Beta Testing

The sample was entirely male, with almost half working in an MDI (Table 3). Participants had a mean age of 38 years (SD=9.23). On average, participants spent just under an hour in the app (mean=58.24 min; SD=62.98) and completed a third (mean=9.11; SD=10.25) of the challenge days. Over half (n=48) of the participants reported a prior episode of mental ill-health and were considered high-risk on the HILDA-derived risk algorithm [36].

#### Preliminary Effectiveness

Although usage data were collected on all participants, only 34 (40.5%) completed follow-up questionnaires. No differences were found on any baseline data collected between responders and nonresponders; however, those responding to follow-up completed significantly more challenges (*t*_54.23_=4.12; *P*<.001), app sessions (*t*_41.62_=3.22; *P*=.002), and active time (*t*_41.49_=3.38; *P*<.002). At 5-week follow-up, the *HeadGear* app was associated with significant reductions in depression symptoms (*t*_30_=2.53; *P*=.02; Cohen *d*=0.39), anxiety symptoms (*t*_30_=2.18; *P*=.04; Cohen *d*=0.38), and overall past month sick days (*t*_28_=2.38; *P*=.02; Cohen *d*=0.22) and increases in self-reported workplace productivity (*t*_28_=−2.09; *P*=.046; Cohen *d*=0.33). Trends toward improvement were found for well-being and mental health sick days, although these did not reach significance (Table 4).

Further analysis was conducted to determine whether improvement in depression and anxiety symptomatology was related to app usage. The results showed that there was a significant association between change in depression symptoms and time spent using app (*F*_1,31_=6.08, *P*=.02; *R*^2^=.164). Similarly, there was also a significant association between change in anxiety (*F*_1,29_=5.35, *P*=.03; *R*^2^=.174) and well-being (*F*_1,30_=4.15, *P*=.049; *R*^2^=.121) and time spent using the app. These results suggested that more time spent using the app was associated with a greater reduction in depression and anxiety symptomatology and a greater improvement in well-being. No other comparisons reached significance. Additional analysis indicated that the change in depression and anxiety symptomatology was not related to participants’ level of risk category or industry type.

#### Feasibility and Feedback

**Figure 3:**
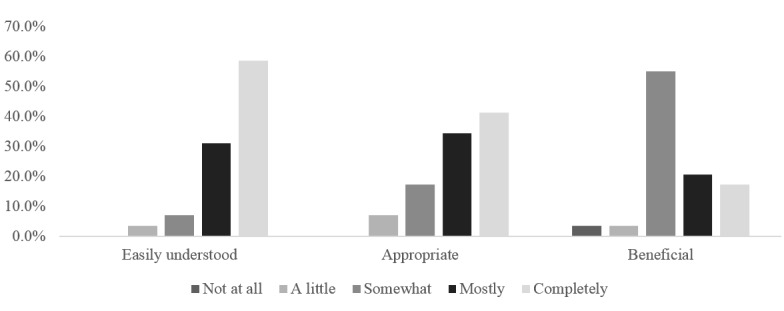
*HeadGear* pilot beta feedback (n=34).

Figure 3 presents the basic feasibility of the program (n=34). Over three quarters (76%) of the respondents found the app to be mostly or completely appropriate for them, over 90% claimed it helped them improve their mental fitness (at least somewhat), and 90% found it mostly or completely understandable. Users were asked about the best and worst features of the app (stability, speed, look and feel, functionality, navigation, content, and other). Content was the most popular feature reported (46%), followed by both look and feel and functionality (23%). Navigation was the most highly ranked issue with the app (23%).

**Table 4:** Effectiveness outcomes. Italics indicates significance at the .05 level.

Outcome measure	Pretrial, mean (SD)	Posttrial, mean (SD)	Significance
Patient Health Questionnaire-9	12.00 (5.93)	9.68 (5.86)	*.02*
5-item World Health Organization Well-Being Index	9.29 (4.26)	10.00 (5.45)	.47
Connor Davidson Resilience Scale	23.57 (7.32)	23.27 (8.13)	.75
2-item Generalized Anxiety Disorder	2.77 (1.61)	2.16 (1.63)	*.04*
Absolute presenteeism^a^	53.79 (28.34)	63.10 (20.20)	*.046*
Sick days past month	2.31 (4.86)	1.24 (3.06)	*.02*
Mental health sick days past month	1.59 (4.87)	0.90 (2.85)	.10

^a^A score of self-reported workplace productivity (higher scores=greater productivity).

Open feedback on the app was generally positive, with mindfulness and value-based goal setting highly regarded:

Improved my focus to make mindfulness a more consistent part of my day.

Great app has really helped me look at all aspects of my life: work, relationships, interests, exercise, diet and mindfulness. This app has helped me manage my anxiety and depression.

However, engagement and personal commitment were consistently raised as issues:

I wasn't able to sustain engagement with it. This was mostly through having some really good days. My mental health is constantly fluctuating. I think the content I saw was really good and I think if I had the time (didn't work so much) and was in a worse way [sic] would've used it more consistently.

Some users reported disengaging from the longer challenges:

Disengaged from longer sessions, feeling like I wasn't acting on set actions without consequence.

While creating time was also an issue:

I didn’t make enough time to complete it,

(You need to) Break up long sessions/ (have) time limited options.

Only 8 respondents wanted to see additional features in the app; these features included a sleep tracker, ability to download and print, rescheduling of reminders (already present in the app), more reminders, a journal space, and longer mindfulness exercises.

## Discussion

### Principal Findings

This study provides both a framework for the development and testing of a new smartphone app intervention, *HeadGear*, and investigates the use and acceptability, along with the feasibility and preliminary effectiveness, of the app in a working population, specifically MDIs. The core features and functionality of the app were developed through a participatory design process, and the content of the app was based on current best available evidence-based theory. The research team encompassed computer engineers, psychiatrists, psychologists, and design (user experience and graphic design) experts allowing for a multidisciplinary approach to development. The pilot testing of the app incorporated a 2-stage process that utilized different samples and different outcomes measures to reflect the progression of the app from alpha to beta testing. Overall, the app was well received in both stages of the pilot testing, and preliminary testing indicated significant improvements in measures of psychopathology and workplace productivity.

The results from this feasibility and efficacy pilot trial suggest that an mHealth app can be an engaging, useful, and acceptable intervention. Across the 2 stages of the study, the majority of the participants acknowledged the utility, helpfulness, and overall ease and acceptability of use of the *HeadGear* app. With regards to preliminary efficacy of the intervention, the results are in line with previous findings that have shown mindfulness and behavioral activation to be effective in the treatment of mood and anxiety disorders [58,59], even in mobile app forms [24]. The dose-effect response seen between level of usage of the *HeadGear* app and improvements in both depression and anxiety symptoms was also encouraging; however, due to high attrition, findings need to be interpreted with caution.

Although improvements in well-being and resilience were found, these findings were not significant. As the sample included both *well* and *unwell* individuals, it is likely to have been underpowered to detect such changes; this underscores the need for a full-scale efficacy trial. Results also indicated that there were significant reductions in absenteeism and increases in worker productivity. This is especially encouraging given medical interventions in isolation have not shown as positive an effect on work-related outcomes when compared with workplace interventions [60]. This finding suggests the utility in incorporating evidence-based interventions in the workplace.

### Strength and Limitations

Despite the positive reviews of stage 1, there was a low level of challenge days completed. This may reflect a number of functionality issues resolved for stage 2 and that the sample’s characteristics were not representative of MDIs (from which this sample was taken and for which the app was designed) as 50% of participants were women. When contrasted with a technically improved iteration and a more representative population (stage 2), there was significantly more engagement with the app. Nevertheless, engendering motivation to complete the program was a concern raised in this review process. Although reasons for disengagement are complex and rarely only due to dissatisfaction [61] and somewhat unsurprising given the unguided nature of the trial [62,63], it does raise some feasibility concerns. O’Brien and Toms [64] suggest that engagement is not static but a process operating over a continuum; therefore, understanding this process more specifically across each of the challenge days might assist in improving adherence, which may be garnered through a larger trial. It was determined in earlier development steps [30,31] that end users were familiar with month-long health endeavors (eg, FebFast, Steptember, Dry July), and this played a role in the selection of the 30-day challenge period. Mobile apps, in general, suffer from poor rates of retention. Overall, 43% of global mobile users were still using apps (at least once) 1 month after download [65]. However, 23% will use an app only once, and only 1 in 3 will use an app at least 11 times [66]. Ultimately, this presents new obstacles in regards to engagement with a mental health and well-being app that need to be considered over the full intervention [67]. Encouragingly, results indicated the more time spent in the app was associated with more positive outcomes on the primary outcome and that participants used the app irrespective of their current symptom level, suggesting it has wider appeal than simply those with heightened symptomatology. Nevertheless, further research is required to better understand ways in which to enhance engagement.

A substantial strength of the study was the development process, which allowed for detailed and systematic analysis of a product in multiple stages of testing. Additionally, the mobile-based delivery of the program holds a number of advantages over traditional methods particularly in MDIs [68]. Despite some limitations to generalizability, the study indicates that the intervention may have value in engaging this difficult-to-reach and at-risk group [69]. Indeed, tailoring the treatment to the feedback received during participatory user research meant that goal-directed and skill-based activities were the predominate focus of the intervention, which is in line with other recommendations for this group [70].

In addition to modest rates of intervention completion, the follow-up rate was also a limitation, and as mentioned, this has implications for the findings. Despite email (stage 1) and SMS text message (stage 2) reminders and incentives for assessment completion, follow-up rates were low compared with the literature [71]. Some reasons postulated for this include the source of recruitment (social media in stage 2), limited exclusion criteria (ie, those who were well may have had less motivation to engage), and a lack of personalized follow-up. However, a key factor which is unique to this trial is onboarding, whereby participants downloaded the app, consented, and subsequently completed baseline within the app. Therefore, users may have had little desire to participate in the trial but simply wanted access to the app. In an attempt to streamline the user experience (avoiding filtering participants through an arduous onboarding, which may lose all but the most conscientious participants), the study may, in fact, have recruited a less research-engaged (though perhaps more real-world) sample. Clearly, alternate and intensive strategies are required, as these low levels of retention raise feasibility concerns for a larger randomized controlled trial (RCT) trial. Additionally, low levels of mental health sick days were reported in the sample. This is unsurprising considering the small size and short follow-up; however, it limits what can be derived from this outcome. Sample size limited further investigation of change in outcomes based on baseline risk category or industry; again, larger RCT studies are required to explore these relationships. An additional limitation is despite targeting MDIs, there was significant interest from non-MDIs, and consequently, the conclusions that can be reached pertaining solely to MDIs are limited; conversely, the app may have wider utility. Finally, and perhaps most importantly, the lack of a control group limits any conclusions that can be made regarding the beneficial impact of this app; an RCT would help to ameliorate the biases inherent in uncontrolled trials.

### Conclusions

The results from this pilot trial suggest that the *HeadGear* app can be an engaging, acceptable, and potentially effective intervention. Although preliminary results were encouraging, noted limitations in the pilot design highlight the need for a full-scale efficacy trial to better understand the utility of smartphone apps in the prevention and treatment of depression symptoms.

## Abbreviations

CBT: cognitive behavioral therapy
CD-RISC: Connor Davidson Resilience Scale
CMD: common mental disorders
eHealth: electronic health
HILDA: Household, Income, and Labor Dynamics in Australia Survey
MDI: male-dominated industries
mHealth: mobile health
MRC: Medical Research Council
PHQ-9: Patient Health Questionnaire-9
RCT: randomized controlled trial
SMS: short message service
UNSW: University of New South Wales
WHO-5: 5-item World Health Organization Well-Being Index
WHO-HPQ: World Health Organization Health and Work Performance Questionnaire
